# Late retirement, early careers, and the aging of U.S. science and engineering professors

**DOI:** 10.1371/journal.pone.0208411

**Published:** 2018-12-26

**Authors:** Navid Ghaffarzadegan, Ran Xu

**Affiliations:** Department of Industrial and Systems Engineering, Virginia Tech, Falls Church, Virginia, United States of America; Aga Khan University, KENYA

## Abstract

Studies of rescuing early-career scientists often take narrow approaches and focus on PhD students or postdoc populations. In a multi-method systems approach, we examine the inter-relations between the two ends of the pipeline and ask: what are the effects of late retirement on aging and hiring in academia? With a simulation model, we postulate that the decline in the retirement rate in academia contributes to the aging pattern through two mechanisms: (a) direct effect: longer stay of established professors, and (b) indirect effect: a hiring decline in tenure-track positions. Late retirement explains more than half of the growth in average age and brings about 20% decline in hiring. We provide empirical evidence based on the natural experimental set-up of the removal of mandatory retirement in the 1990s.

## Introduction

Newly minted PhDs in science and engineering in US face major career challenges including low rates of hiring in academia, skyrocketing population of postdoctoral researchers (postdocs), and low chances of receiving research funding [1–3]. Abundant studies have focused on the population of PhDs and postdocs to study supporting mechanisms that can help their career, an example being recent suggestions of the *National Academies of Science*, *Engineering*, *and Medicine* on capping postdoc duration at three years, training them for industry positions, or allocating funding for career development training of postdocs [4]. On a separate thread of policy research, it is also noted that the average age of the U.S. science workforce has been increasing over the past two decades [5]. This growing pattern (Table 1) has also been observed among faculty members in different academic fields [6–9], and the median age of professors has now surpassed all other occupational groups [10].

**Table 1:** Changes in age measures of faculty members between 1995 and 2010.

	Average age			Age distribution measures				
	Among all tenure track and tenured faculty	Only among tenure track	Only among tenured	50 years or older	55 years or older	60 years or older	65 years or older	70 years or older
**1995**	48.05	37.02	50.03	45%	25%	12%	4%	1%
**2010**	50.53	38.83	53.74	54%	39%	24%	11%	3%

Source: NSF’s Survey of Doctorate Recipients (SDR)

The career problems of newly minted PhDs and the retirement trends of senior faculty have been studied disjointly (exception [11]). On the problem of early career scientists, study examples include examination of postdocs’ productivity, postdoc duration, and effects of postdoc training. On the other hand, on retirement, effects of elimination of mandatory retirement on faculty members’ choices to postpone retirement have been well documented [12–15]. Few studies have systematically examined how late retirement affects long-term aging trends and hiring of early career scientists. An exception is Larson and Gomez Diaz’s study which develops a queueing model of faculty workforce and uses data from a U.S. institution to estimate effects of late retirement on hiring new faculty [11]. Many other studies assume hiring is independent from retirement (e.g., [5]).

There is no doubt that senior faculty members play a critical role in mentoring junior researchers, provide invaluable service to their institutions and research communities, and are pivotal in experience transition and network connections. However, a continuously aging faculty also poses many institutional challenges, such as greater uncertainty in research productivity, hyper-competition for early career scientists seeking research funding, considerable financial burdens in terms of salary and benefit obligations, and an increasing need to raise student tuition or request federal and state subsidies [16–22]. Trying to develop effective policies to help early career scientists with a sole focus on the population of PhDs and/or postdocs, and without looking at the changing dynamics of established faculties would be an example of a narrow boundary analysis.

To foster evidence-based policy discussions regarding the aging pattern of U.S. faculty, we report on two complimentary studies. First is a simulation model of U.S. tenure-track faculty, from hiring to retirement or change in job, developed to explore aging patterns and hiring rates in academia. The model is a differential-equation based system dynamics model which represents an aging chain of US faculty, and examines change in age distributions as affected by change in retirement tendencies. The model is validated by replicating the past data. Second, we report a quasi-experimental analysis where we employ a statistical approach (difference-in-differences) to test the effects of the elimination of mandatory retirement. We utilize the opportunity that different states in the US implemented the removal of mandatory retirement in different years, many prior to becoming a federal law. Comparing and contrasting hiring and average age in these institutions in different time periods provide insights into the effects of late retirement on US faculty workforce. The second study confirms conclusions of the simulation model.

Overall, this study offers a different perspective to explain a source of problems of early career scientists, and the interconnections in the science workforce, stressing the inadequacy of looking at the young faculty population in isolation. The study shows the magnified effects of late retirement on the growth in average academic age through two mechanisms—a longer stay of established professors and a decline in the hiring rate of newly minted PhDs. We discuss our findings and offer several policy implications to help early career scientists in the U.S.

## Materials and methods

### Study 1: Simulation model

#### Data

We use the Survey of Doctorate Recipients (SDR), a longitudinal survey of the science workforce population with doctoral degrees in science, engineering, and health earned in the U.S (http://www.nsf.gov/statistics/srvydoctorates/). The SDR is sponsored by the National Science Foundation (NSF), and is usually administered every two years. The sampling frame is the Survey of Earned Doctorates (SED), an annual census of individuals receiving a research doctorate. Once in the sample, individuals are surveyed repeatedly until age 76. The sample is refreshed with new doctorate recipients at each wave. The SDR includes rich information of doctoral recipients such as date of birth, educational history, employment status, field of degree, geographic place of employment, occupation, labor force status, race/ethnicity, salary, sex, and many others. The SDR’s sample size is one of its strengths. For example, the overall sample size for the 2010 survey was more than 30,000, with the overall sampling rate around 5.2%. Response rates are considered to be good; for example, 79.8% of those surveyed completed it in 2010. Several other studies have used this data set (e.g., [5]) to study the population characteristics of the science workforce.

Our study sample includes U.S. science and engineering doctorates employed as tenure- track and tenured faculty members in academia. Scientific fields of the faculty include the life sciences (biology, medical science, etc.), physical sciences (chemistry, physics, astronomy, and geology), engineering, computer science and mathematics, and social science (economics, psychology, etc.). In study 1, we use eight survey waves conducted from 1995 through 2010. We primarily used 1995–2010 survey data of tenured and tenure-track faculty to calibrate our simulation model. The data contains 65,208 observations on approximately 19,518 faculty aged 76 or less, with an average of 3.3 observations per sample member. SDR survey weights, provided by the NSF, are used in our analysis to adjust for attrition bias, resulting in more representative data of the population. To calibrate our simulation model, we use the SDR survey to extract detailed information such as the faculty members’ age distribution, hiring rate, and exit rate at each age.

#### Model

**Fig 1:**
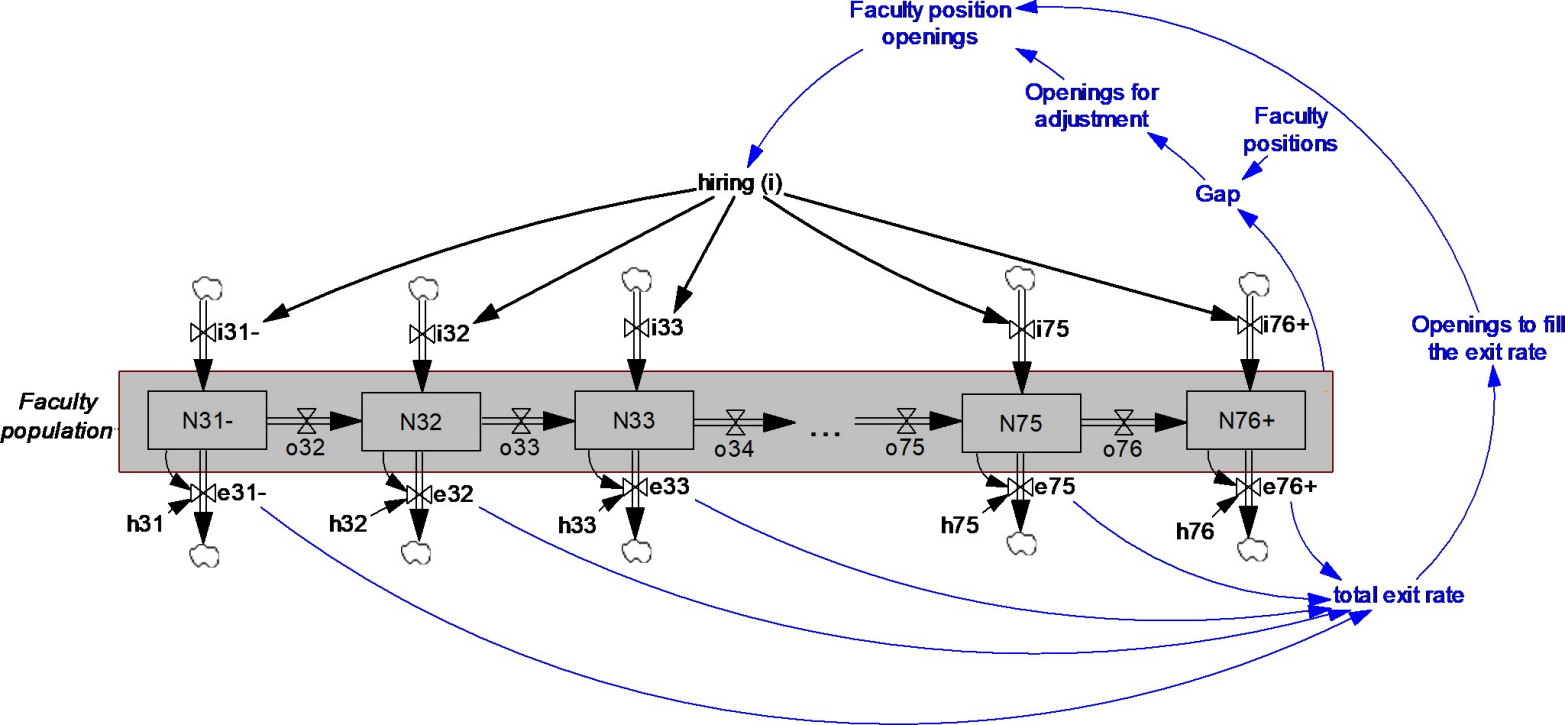
A simple representation of a stock-flow model of faculty members. Note: the value for *faculty positions* is not necessarily constant; numbers depict age; N: population; i: hiring inflow; e: exit rate; o: aging; h: hazard rate of attrition.

We extend a previously published simulation model [5] by formulating hiring PhD workforce in faculty positions as affected by limited university capacities. This helps demonstrate where the variation of our results from previous studies emerges. We will later conduct an independent empirical examination to depict fidelity of our model predications. Fig 1 shows a conceptual diagram of the simulation model. The large center rectangle represents professors employed in tenure-track or tenured positions in academia. Inflows are hiring at different ages and outflows are exit rates at different ages that include retirement, tenure denial, or attrition for other reasons such as taking a job outside academia. The number of new PhD graduates, postdocs, or similar populations that could be interested in academia, as well as academic position openings, affect the inflow. The latter is formulated as the sum of openings to fill positions of people who exit academia (in the Fig 1, *Openings to fill the exit rate*) and additional new positions that open due to an increase in university capacity (in the Fig 1, *Openings for adjustment*).

We simulate the model from 1995 to 2010. For each iteration, the model generates value for each variable and transition rates between them endogenously. The only measure we change exogenously over time in the simulation is the exit rate and total faculty positions. The total number of faculty positions in the base runs is set based on 1995–2010 data. We simulate this model using the following inputs: age distribution of faculty, exit rate, and hiring rate, all in 1995.

To model aging, the central rectangle in the conceptual model (Fig 1) is formulated following the same logic described in reference [5]. If we represent the total number of people in academia with *N*_*total*_, this population includes faculty of different ages. We use 46 stocks that represent faculty members aged 32–75 (44 stocks), one stock for 31 years old and younger, and one stock for 76 years old and older. If we define *N*_*a*_(t) as the number of individuals employed as faculty members at age *a* at time *t*, the total faculty population, *N*_*total*_, is:

$$N_{total}(t)=\sum_{a=31}^{76}N_{a}(t)$$ (1)

People age through the pipeline, moving from the left side of the figure to the right. For each age category, *N*_*a*_(t), there is an inflow that represents new hires, and an outflow that represents the retire/exit rate of that specific age.

As stated, in this model, hiring is formulated endogenously, which makes it different from Blau & Weinberg’s (2017) model [5]. Specifically, the total number of faculty position openings each year is comprised of (i) openings for adjustment, which are new positions due to an increase in university capacity, and (ii) openings to fill the exit rate, which are the positions of ones who have left.

We define *h*_*a*_ as the annual hazard rate of attrition for faculty with age *a*. Note that this hazard rate includes retirement, transition to a non-science job, and transition to a science but non-faculty job. In each time period, *i*_*a*_ is the number of faculty hired at age *a*, and *o*_*a*_ is the rate at which people are aging from *a-1* years old to *a* years old. Age-specific transition can then be characterized as

$$N_{a}(T)=\int_{t=1995}^{t=T}[i_{a}(t)-h_{a}(t)∙N_{a}(t)+o_{a}(t)-o_{a+1}(t)]dt+N_{a}(1995)$$ (2)

*i*_*a*_ is estimated by the total hiring *i*_*total*_ multiplied by *p*(*hiring*, *a*), the proportion of new hires at *a* years old in 1995. Data show that the age distribution of new hires does not change much during our analysis. Thus we set:

$$i_{a}(t)=p(hiring,a)∙i_{total}(t)$$ (3)

For endogenous formulation of hiring, we have

$$i_{total}(t)=[\tilde{N}(t)-N(t)]+\sum_{a=31}^{76}N_{a}(t)∙h_{a}(t)$$ (4)

The first term, $\left[\tilde{N}(t)-N(t)\right]$, is the difference between capacity and the total number of faculty members, which represents the faculty capacity gap. The second term, $\sum_{a=31}^{76}N_{a}(t)∙h_{a}(t)$, is the total number of faculty members leaving academia at all ages at time *t*. If $\tilde{N}=N$, it represents a steady state for the number of faculty members, and hiring will only replace the exit rate ($i_{total}(t)=\sum_{a=31}^{76}N_{a}(t)∙h_{a}(t)$). For $\tilde{N}$, we use the past data and assume a +1.3% annual growth rate (the average growth rate in 1995–2010). Our main counter-factual run in which the only difference with the base run is the constant retirement rate is referred as scenario s1 in the paper. We conduct a sensitivity analysis for growth rates of 0.65% to 1.95% in $\tilde{N}$, results represented as scenarios s2 and s3 in the paper. We also simulate for conditions when increase in hiring presumably results in hiring older faculties. Scenarios s4 and s5 in the paper represent average hiring age of one and two years older than the base run. These conditions are implemented by shifting the hiring distribution to the right, one or two years older, respectively.

We estimate the annual hazard rates of attrition by looking at the difference between net changes in each stock variable in two consecutive surveys adjusted for hiring rates and divided by 2. We use the data from 1995–1999 and 2008–2010 to estimate the hazard rates of attrition in our simulation model; for the period between, we linearly interpolate.

Finally, if we focus on the outflow of each stock every year, *h*_*a*_(*t*) proportion of faculty leave academia, and (1-*h*_*a*_(*t*)) proportion of them age while still employed. Thus:

$$o_{a+1}(t)=\left(1-h_{a}(t)\right)∙N_{a}(t).$$ (5)

We simulate the model from 1995 to 2010, using the Vensim DSS software package, which is generally a good package for differential equation-based models (including system dynamics models). The integration type is Euler. Our time unit is one year which provides a more systematic comparison of the model with previous models (For numerical estimations, we set dt = 0.125 of time unit and ensured that the results are robust for smaller values of dt). We also kept the model details on a level at which we can see age distribution (not just average age) and then compare our results with previous models. The model and its inputs are provided as supplementary material (S4 File). For readers interested in potential extensions of the model for future work, and building more complex system dynamics models of a faculty workforce, we report causal loop diagrams of several dynamic hypotheses that can influence the process of aging and hiring in academia (simple vs. medium complexity [this paper] vs. high complexity), depicted in Figures A-C in S3 File respectively. In this paper, our intention was not to incorporate all of these mechanisms, however, the provided map in Figure C in S3 File has been very useful in exploring potential hypotheses in our initial stages of modeling.

### Study 2: Statistical analysis

#### Data

We use the same data source as used in the simulation model. In study 2 we included 1983–2010 survey data of tenured and tenure-track faculty to study the long-term age trend, which contains a total of 109,499 observations.

#### Identification strategy

Although most institutions had enforced mandatory retirement by December 31, 1993, some schools had eliminated mandatory retirement before that date, and most of these decisions were driven by a state law that prohibited mandatory retirement [12, 23]. As such, there are two subgroups of institutions: those that did not eliminate the mandatory retirement policy until the federal law took effect in 1994 (late uncap), and those that eliminated mandatory retirement policy somewhat earlier (around 1990s) (early uncap). By comparing early uncapped schools with late uncapped schools prior to 1994 we can estimate the effect of elimination of mandatory retirement. The early uncapped group includes all institutions in Wisconsin, Maine, Utah, Montana, and Nevada, and all public institutions in Alabama, Arizona, Connecticut, Florida, Idaho, Louisiana, New Hampshire, New York, Texas, Virginia, and Wyoming. If the intervention of uncapping has any effect on the average age, we expect to see an increase in the average age of early uncapped schools prior to 1994.

To empirically test how the elimination of mandatory retirement has contributed to the increase in the age of tenure-track faculty, we use an individual-level SDR data set and faculty age as our main outcome. We employ two identification strategies. First, we conduct a difference-in-differences analysis. We compare the average faculty age of early uncapped schools with that of late uncapped schools in a difference-in-differences analysis. We use SDR data from 1983–1993 in which early uncapped schools (treatment group) have eliminated the mandatory retirement around 1990, and late uncapped schools (comparison group) have not eliminated the policy in the time horizon. For most of the early uncapped schools the mandatory retirement policies were eliminated around 1988 to 1991 [23]. As we do not have observations within this period in our sample, we define our treatment period as after 1990. Here the deviation from the trend in the comparison group will reflect other hard-to-observe factors (e.g., the economy, or other higher education reforms) that may have influenced faculty age in the absence of the elimination of mandatory retirement. We also controlled for the year trend, faculty characteristics such as rank, field, salary, gender, race, marital status and citizenship, and so forth, as well as characteristics of the institution where faculty are employed such as the type of institution, whether it is privately controlled, and Carnegie classification.

Second we look at effects of years-since-intervention, i.e., treatment as dosage. In the previous analysis we could only use SDR data up to 1993 because late uncapped schools had eliminated mandatory retirement from 1994 onwards. Here we consider treatment as dosage—that is, we define the treatment variable as the number of years a school has eliminated the mandatory retirement policy. This method allows us to include more years of data in our analysis (1983–2010), and we can statistically compare the faculty age of a school that has eliminated mandatory retirement for a certain number of years. In addition, because schools in the two groups (late and early uncapped) eliminated mandatory retirement at different time points, we also gain the statistical power to estimate the effect of elimination of mandatory retirement within the same year. In this analysis we include year fixed effects and other control variables as in the previous model. Note that in both analyses we have also controlled for institutional fixed effects or use institution or state level clustered standard error as alternative specifications, which our results are robust to.

Finally, to estimate the effect of elimination of mandatory retirement on the number of new hires, we construct an institution-level data set and use the number of new hires for an institution in a year as the main outcome, employing a difference-in-differences analysis. Since hiring data were available from 1993, we consider the removal of mandatory retirement due to the federal law as the exogenous intervention. This, in contrast to the previous analysis, makes the late uncapped schools the treatment group, and the early uncapped schools the comparison group. The assumption is that the hiring of early uncapped schools is arguably much less affected by the elimination of mandatory retirement at the federal level from 1994, as such schools had already eliminated this policy considerably earlier than 1994. Thus, the additional difference in the number of new hires between the schools in the two groups after the elimination of mandatory retirement on the federal level can be attributed to the effect of the federal policy. As some lags might exist in the impact of the elimination of mandatory retirement on new hires (i.e., it takes time for faculty positions to saturate and thus decrease the number of new hires), we test how our estimates are sensitive to different specifications of the post-policy period. Other control variables are the same as before, with the exception of using aggregate measures of faculty characteristics (e.g., number of assistant professors, number of females, and so forth.) at the institutional level. Note that here the institutional-level data is aggregated using individual-level data with weights. One potential limitation of this approach is that the SDR sample might not be fully representative on the institutional or regional level. However, the difference-in-differences estimates would still provide reasonable estimates for the magnitude and direction of the effect under the assumption that representativeness of the sample on the institutional level does not vary across years.

## Results

### Descriptive trends

**Fig 2:**
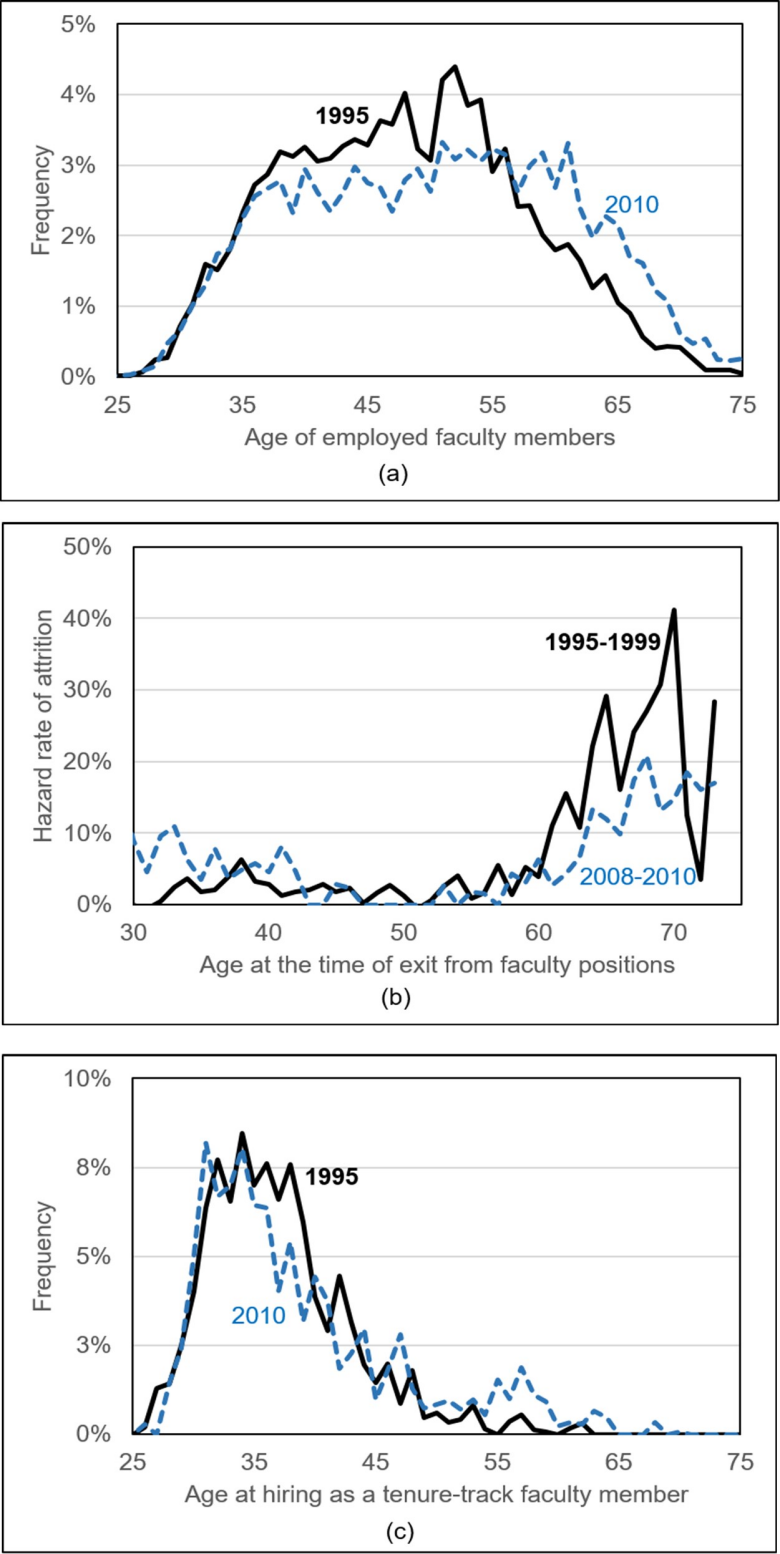
Age distribution pattern in US academia. **(a) Age distribution of faculty in 1995 and 2010. (b) Annual hazard rates of attrition based on 1995–1999 surveys and 2008–2010 surveys. (c) Age distribution of new tenure-track hires in 1995 and 2010.** Note: we define a new faculty member as a tenure-track faculty member hired within two years when observed.

First we look at some important descriptive trends. Fig 2 shows age distribution details of faculty members, new hires, and exit rate in 1995 and 2010. In Fig 2A, we observe a shift in the age distribution toward the right side (older ages). There is a non-negligible decline in the share of faculty aged 35–55 years old along with a significant increase in the share of faculty aged 55 and older.

The two main determinants of the faculty age distribution include: (i) hiring rate and age distribution at hiring, and (ii) exit rate and age distribution when exiting. We look at the entrance and exit rate of faculty members to make more sense of the growing age pattern. Fig 2B shows the age distribution at the time of exit from faculty positions. In the 1995–1999 period, the exit rate was very low before age 60. After age 60, it sharply increased, and spiked at around age 70. However, in recent years, the increase in exit rate after age 60 has greatly lessened, and the spikes around age 70 disappear as well. This pattern is consistent with the end of mandatory retirement at age 70 in universities and the assumption that the effect on people’s behavior to retire was delayed [5]. Fig 2C depicts the age distribution of new hires for tenure-track positions in academia in 1995 and 2010, respectively. Change in the hiring distribution is minimal, and it appears that the majority of new hires are in the age range of 30–40.

### Simulation results

**Fig 3:**
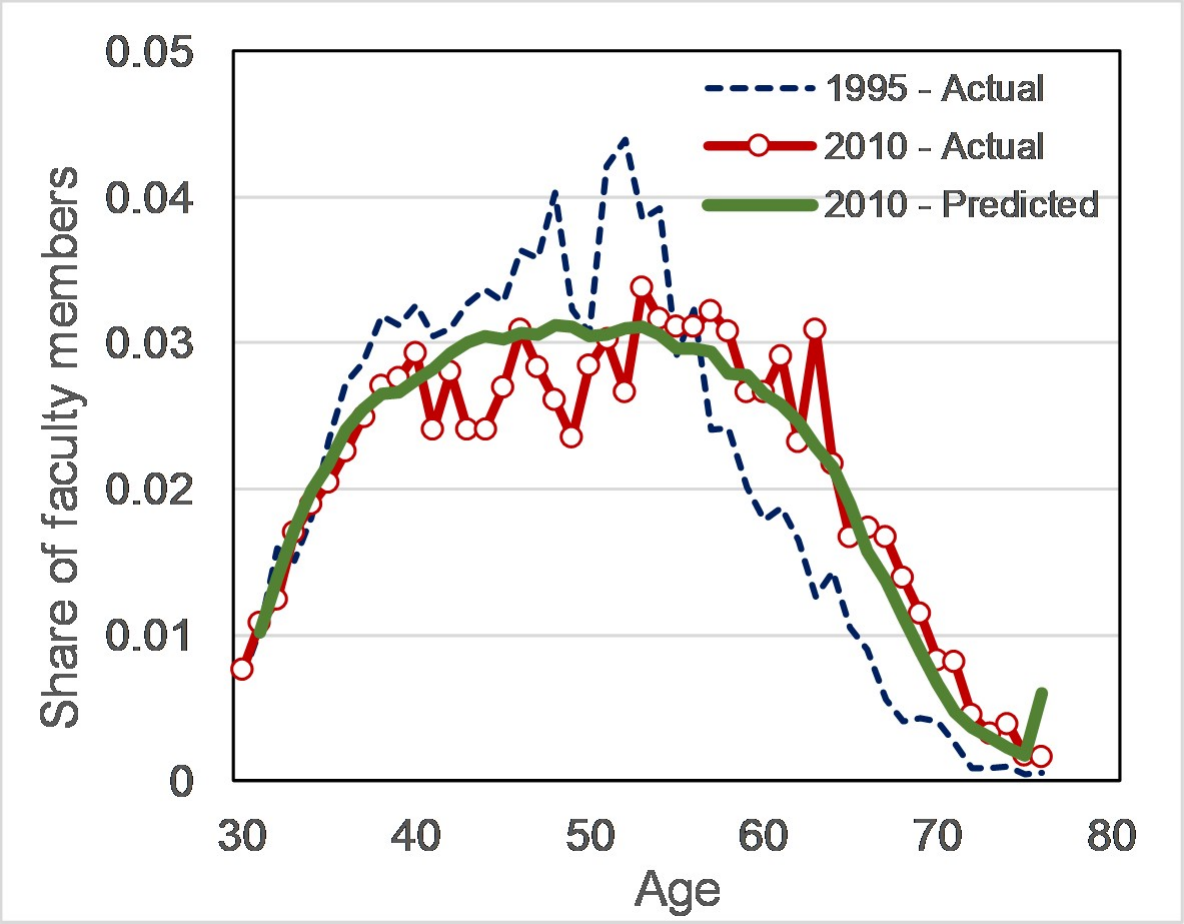
The age distribution of faculty members in 1995 and 2010 and the model’s prediction for 2010 (the correlation between predicted data and observed data in 2010 is 0.95).

We first test the model’s results for 2010 and show that the model successfully replicates the age distribution in 2010 data (Fig 3). Having built confidence in the model, we conduct a counter-factual analysis by setting hazard rates constant, and equal to the estimated values for 1995–1999 to test what would have been average age and hiring rate if the retirement rate was unchanged (Table 2, counter-factual test, scenario s1).The model estimates average age of 48.8 (in comparison to the base run of 50.3), and hiring rate of 12.8 thousands (base run of 10.7 thousands) in 2010. In other words, late retirement has caused average age growth of 1.5 years and 16.4% decline in hiring. One argument would be that average age of new hires or university capacity growth would not remain the same if retirement was kept constant. To check robustness of the model to potential changes in other parameters, we run the model for four different scenarios representing changes in university capacities and average age of hiring in reasonable ranges (Table 2, scenarios s2-s5). The results are qualitatively robust depicting average age growth of about 1.1 years (51% of total change in age) and 15.5% decline in hiring across all scenarios.

**Table 2:** Simulation results.

	Base run	Counter-factual tests (constant retirement rate)				
		s1	s2	s3	s4	s5
Average age						
1995	48.1	48.1	48.1	48.1	48.1	48.1
2005	49.9	49.4	49.5	49.3	49.8	50.2
2010	50.3	48.8	49.3	48.4	49.4	50.0
Hiring [thousands]						
1995	6.3	6.3	6.3	6.3	6.3	6.3
2005	7.8	8.9	8.3	9.5	9.0	9.0
2010	10.7	12.8	10.4	15.1	12.9	13.0

Note: In all counterfactual tests, retirement rate is fixed to the same value as 1995–1999. Scenarios: s1: University growth rate (*r*) of 1.3% (the same as base run) and average age of new hires (*a*) of the same as base run (38.3 years old); s2: the same as s1 but r = 0.65%; s3: the same as s1 but r = 1.95%; s4: the same as s1 but a = 1 years older than base run; s5: the same as s1 but a = 2 years older.

Our simulation study suggests that the decline in retirement rate of older faculty is one of the main drivers of the aging pattern for U.S. faculty for two reasons: (i) it has the direct effect of keeping more elderlies in academia, which increases the share of older scientists among all faculty; and (ii) as the exit rate decreases, it also decreases the number of openings and limits the number of younger scientists entering the faculty workforce. Additional analysis of the model is provided in the supplementary including sensitivity analysis of our results to the assumption of different values of hiring elasticity (Table A and Figure A in S1 File) and forecasting the trend (Figure A in S2 File). In order to forecast the future trend, we run the model until 2025 to estimate the future trend of aging. In the most reasonable scenarios of 1%–2% annual growth in university capacities, the average age of faculty members is predicted to reach a steady state of 50.9–51.7 years in 2025.

The modeling process and simulation results uncover the system structure and potential causal mechanisms, and help perform counterfactual analysis. While the model is consistent with a previous study [5], we agree that the insights should be interpreted carefully and within the model boundary and assumptions. For a precise estimation of the order of effects with confidence intervals, we pursue a statistical analysis as a supplementary study. This also helps to further explore validity of the simulation model outcomes.

### Statistical results

Before the 1980s, mandatory retirement was almost universal for tenure-track faculty. Although mandatory retirement at age 70 for most workers was outlawed by the 1986 amendments to the Age Discrimination in Employment Act (ADEA), tenure-track faculty in post-secondary institutions were granted a temporary exemption from the law, as colleges and universities argued that mandatory retirement was needed to maintain a steady inflow of young faculty and promote the hiring of women and minorities [12]. Following a review in 1991, however, Congress allowed the exemption to expire, and mandatory retirement of tenure-track faculty was eliminated on January 1, 1994. Note that some universities and colleges (mostly public institutions) had already eliminated mandatory retirement before that date as some state laws prohibited mandatory retirement. We utilize variation in timing of the elimination of mandatory retirement at age 70 for U.S. tenured faculty in different states [12–15, 23].

**Fig 4:**
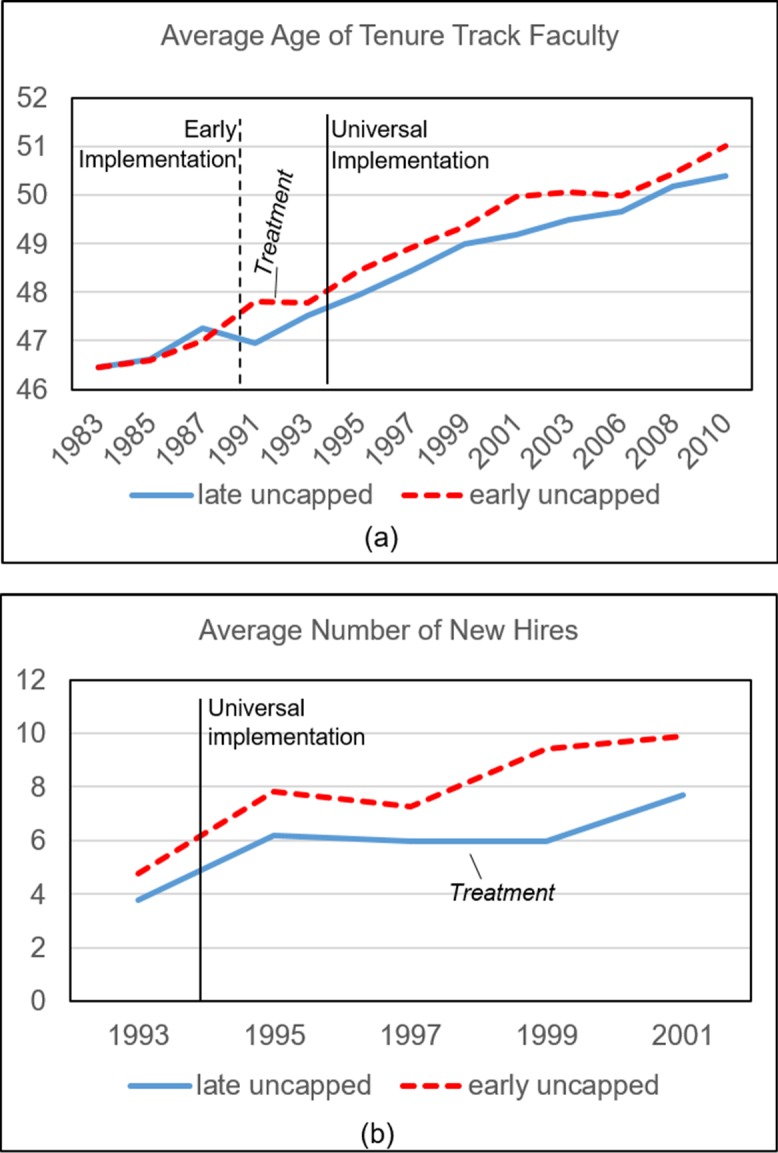
**(a) The trend of average age of tenure-track faculty from 1983 to 2010 by type of institution (whether they eliminated mandatory retirement early or late); (b) the trend of the average number of new hires for each institution from 1993 to 2001 by type of institution.** Note: Black solid line (universal implementation) indicates the time of elimination of mandatory retirement on the federal level for all schools, black dashed line (early implementation) indicates the elimination of mandatory retirement on the state level for early uncapped schools.

First we check the overall trends in Fig 4A and 4B. Fig 4A is consistent with our hypothesis. Early uncapped schools and late uncapped schools share similar trends of age growth in faculty age before 1987. However, early uncapped schools enjoyed a larger growth after 1990 compared to the late uncapped schools—that is, immediately after they eliminated the mandatory retirement policy. The average age of tenure-track faculty in late uncapped schools has increased considerably, as well around the period of the elimination of mandatory retirement on the federal level, and schools in both groups have maintained a similar increasing trend of faculty age since 1993. However, the difference of the average age between the two groups has kept steady at about 0.5 years, possibly due to the fact that faculty age has been continuously increasing, and the system has not reached the steady state yet.

Fig 4B descriptively illustrates how the estimated average number of new hires (defined as the average number of faculty hired per year for an institution within two years at the time of data collection) changed over years. Hiring data are available after 1993. Given that prior to 1993 some universities have already implemented uncapping, we expect that universities which delayed uncapping until federal requirement of 1994 to show a delayed decrease in hiring. In other words late uncapped should start showing a decline in hiring after 1994 (note that we count the new hire as 0 for institutions with no new hires in a year in our sample, which consist of more than 50% of the observations in the final institution-year level data). Fig 4B shows that schools in both groups have similar new-hire trends from 1993 (the earliest data available) to 1997, after which the late uncapped schools hired considerably fewer faculty when compared to early uncapped schools, suggesting a possible lagged effect of the elimination of mandatory retirement on number of new hires.

Given the trends, we now conduct a more systematic examination. Table 3 shows the regression results for the impact of the elimination of mandatory retirement on the age of tenure-track and tenured faculty based on four models with different identification strategies and different time period of analysis. All of the models in Table 3 explained a significant portion of the variance in the age of faculty, with R-squared ranging from 0.45 to 0.50 (F-test for all of the 4 models are significant, p < .001). Models 1 and 2 focus on the time period of 1983–1993 when uncapping retirement was not mandatory and was only implemented in some states (treatment group: “Uncap”). Results of our difference-in-differences analysis (specifically, the coefficient for “Post-1990*Uncap”) show that the elimination of mandatory retirement is associated with about 0.5 years increase in average faculty age (p < .001). Compared with the predicted increase in faculty age from 1983 to 1993 for schools that implemented mandatory uncapping (~1.5 years), we estimate that the elimination of mandatory retirement explains 31%–37% (i.e., (0.461/1.5 in Model 2 and 0.551/1.5 in Model 1) of the growth in faculty age in that specific time period. Since the time period of three years might not be enough to observe the full-range effect, we expect the effect to be underestimated.

**Table 3:** Regression results for the impact of elimination of the mandatory retirement policy on the age of tenure-track faculty.

	Model 1	Model 2	Model 3	Model 4
Post-1990	-0.718***	-0.763***		
	(0.210)	(0.202)		
Uncap	0.121	-1.373		
	(0.122)	(2.281)		
Post-1990* Uncap	0.551***	0.461**		
	(0.166)	(0.163)		
Year since Uncap			0.113***	0.074*
			(0.016)	(0.036)
Control for Year Effect	Yes	Yes	Yes	Yes
Faculty Characteristics	Yes	Yes	Yes	Yes
Institution Characteristics	Yes		Yes	
Institution Fixed Effects		Yes		Yes
Time Frame	1983–1993	1983–1993	1983–2010	1983–2010
Observations	38,742	39,414	103,485	104,622
R-Squared	0.451	0.513	0.456	0.508
Adjusted R-Squared	0.447	0.492	0.456	0.498

^+^p < .1.

*p < .05.

**p < .01.

***p < .001.

Models 3 and 4 in Table 3 report results from another identification strategy—a continuous treatment variable representing the number of years that mandatory retirement has been eliminated. This identification helps us to look for a longer time period of 1983–2010 and examine the long term effect. The treatment effect (the coefficient “Year since Uncap”) is positively significant. An additional year of the elimination of mandatory retirement is associated with an increase of 0.07 to 0.11 years in faculty age. Compared with the predicted increase in faculty age between 1995 and 2010 (~2.56 years), the elimination of mandatory retirement explains 43%–66% (i.e., 0.074*15/2.56 in Model 4 and 0.113*15/2.56 in Model 3) of the growth in faculty age from 1995–2010, which is consistent with the results from our simulation model.

Table 4 shows regression results for the impact of the elimination of mandatory retirement on the number of new hires at the institution level. Hiring data were available from 1993 and many university had implemented uncapping retirement due to their state laws by then. Thus, we compare the hiring of these universities with universities which only implemented removal of mandatory retirement after 1993 due to the federal law (treatment group “F. Uncap”) in a difference-in-differences analysis. All of the models in Table 4 explained a significant portion of the variance in the number of hiring, with R-squared ranging from 0.61 to 0.63 (F-test for all of the 4 models are significant, p < .001). Models 1 and 2 explore short-term immediate effects (post 1994) and models 3 and 4 look at a potentially delayed/lagged effect (post 1998) of uncapping on hiring. The results of Models 1 and 2 (coefficient for “Post-1994* F. Uncap”) show that the policy is associated with about 1.2 fewer new hires on average for each school in each year (p < .05). Models 3 and 4 provide a stronger support (coefficient for “Post-1998* F. Uncap”) and show that uncapping is associated with 1.3–1.9 fewer new hires on average for each school in each year (p < .01). When compared with the predicted average number of hires post-policy for schools that implemented mandatory uncapping after 1993 (about 6.2 faculty), it translates to 16%–23% (i.e., 1.2/(6.2+1.2) in Model 2 and 1.9/(6.2+1.9) in Model 3) reduction in hiring due to the elimination of mandatory retirement.

**Table 4:** Regression results for the impact of elimination of the mandatory retirement policy on the number of new hires.

	Model 1	Model 2	Model 3	Model 4
Post-1994	3.381***	3.204***		
	(0.864)	(0.840)		
Post-1998			0.550	-0.092
			(0.669)	(0.608)
F. Uncap	0.779	0.911^+^	0.351	0.456
	(0.532)	(0.552)	(0.321)	(0.331)
Post-1994* F. Uncap	-1.177^+^	-1.223*		
	(0.602)	(0.608)		
Post-1998* F. Uncap			-1.867**	-1.341**
			(0.600)	(0.497)
Control for Year Effect	Yes	Yes	Yes	Yes
Faculty Characteristics	Yes	Yes	Yes	Yes
Institution Characteristics	Yes	Yes	Yes	Yes
Time Frame	1993–1999	1993–2001	1993–1999	1993–2001
Observations	4,978	6,217	4,978	6,217
R-Squared	0.611	0.633	0.611	0.634
Adjusted R-Squared	0.607	0.630	0.607	0.630

^+^p < .1.

*p < .05.

**p < .01.

***p < .001.

## Discussion

Our study observes the continuous aging pattern of U.S. faculty and identifies the decline in the retirement rate of older faculty as one of the main factors contributing to the aging of U.S. faculty members. The decline in the retirement rate of U.S. faculty was largely induced by the elimination of mandatory retirement on the federal level in 1994, which eliminated the mandatory retirement at age 70 for all U.S. tenured faculty [23, 24].

In our multi-method study, which included a simulation model and a statistical analysis, we show that this late retirement can explain about half of the growth of the average age of faculty members. We argue that, potentially, the effect of late retirement on aging is multifold for the following reasons: (i) it has the direct effect of keeping more elderlies in academia, which increases the share of older scientists among all faculty, and (ii) as the exit rate decreases, it also decreases the number of faculty openings and limits the number of younger scientists entering into the academic workforce. We estimate that if universities did not experience change in retirement age, the hiring rate would have been about 20 percent more.

Our study provides new insights into the debates about aging of the science workforce especially in academia. The majority of research and policy debates addressing challenges that early career scientists face are focused on postdoctoral positions [1, 4, 25]. Such narrow focus has led to efforts to mitigate symptoms. Here we see that the rate at which senior faculties retire has a considerable effect on the careers of young faculty members. Furthermore, most studies in the area of retirement in academia look at the effects of aging in the science workforce rather than the causes [16–19, 20]. Our approach and results are different from a previous study that estimated a much smaller effect of late retirement on aging [5]. The main reasons for the difference are inclusion of capacity restraints on the demand side (universities) and the indirect effect of late retirement on hiring. Since our analysis is specifically designed for PhD employed in tenure-track or tenured positions in academia where total available positions are very limited and has been only slightly increasing during the past decades the assumptions are reasonable.

There are several policy implications. From a science policy perspective, this increase in age has various implications on scientific productivity and innovation, as well as management of the science workforce [16, 21]. Furthermore, there is abundant evidence that early career scientists are facing major problems, waiting in postdoc positions for a long time [26], and their likelihood of landing tenure-track positions is as low as 16% in some fields, such as biomedical sciences [3]. Our results suggest that policies designed to solve such problems of early career scientists might have been ignoring the important variable of senior faculty member retirement [27]. At the institutional level, incentives for early retirement can include financial and non-financial mechanisms. One potential institutional policy implication would be to develop post-tenure positions in which senior faculty members can retire from tenured positions, but engage in specific activities that they enjoy, which are helpful to the research community, such as mentoring young junior faculty members [28].

This study has several limitations which introduces future avenues of research. First, while the SDR data set used in this study is nationally representative, it does not represent the population outside the United States, thus we do not know if the same trend occurs across the world. Second, in the statistical analysis we estimate the causal effect of elimination of mandatory retirement by utilizing the time differences of the implementation of the policy between different states. However, such time differences are relatively small and we should be cautious when drawing conclusions about the long term effect of such policies. Future research may compare United States with regions or countries that have never adopted such a policy (e.g. China) to study the long term effect of the policy. Finally, while our simulation model captures the key dynamics of the late retirement of faculty on aging pattern, we did not explore all possible mechanisms that can influence the process of aging and hiring in academia. Several of these mechanisms are presented in Figure C in S3 File. In future studies, it would be interesting to extend the model to examine optimal academia replacement rates. Application of different mathematical modeling techniques has proved to be fruitful for analyzing science workforce [29]. New methodological advances in system dynamics modeling and statistical analysis have provided more opportunities to utilize rich quantitative data for building accurate models in response to today problems [30]. Adding demographic variables such as the effect of baby boomers or the immigration pattern on a faculty population would bring interesting viewpoints with policy implications. Given the importance of the aging pattern, as well the potential effects of late retirement on young faculty members’ career—including the increasing number of temporary academic positions [7, 31]—we invite the development of more sophisticated models to study the effects of late retirement on academia. Such models would examine not just the growth in average age, but the productivity of the entire enterprise.

Finally, we acknowledge that established senior faculty members are pivotal to the growth and success of academia through invaluable service and mentorship [32]. We would like to clarify that the point here is not to prevent academic institutions from such a resource. We also don’t mean that higher retirement rate is a “silver bullet” and will solve PhD employment issues. The effect would be considerable, but still not enough considering the rate at which PhD students are graduated and the fraction interested in academic positions [2, 3, 26]. Our point is to emphasize that in order to better understand the aging pattern and low hiring rates, one should look more carefully at the magnified effects of late retirement on academia. Ideally, policy debates should move toward finding solutions that will help academia benefit from experiences of established faculty members while also providing the younger population with more frequent tenure-track positions.

## Supporting information

S1 File Sensitivity analysis. The file documents sensitivity analysis of the simulation model.
(DOCX)

S2 File Future trends analysis. This file includes an additional simulation analysis depicting the future aging trends.
(DOCX)

S3 File Conceptual models. The file includes different conceptual models of aging of university professors with varying complexity.
(DOCX)

S4 File Model and data. The zipped folder includes the simulation model in Vensim DSS (AgeModel-V6.mdl), the data file for the simulation model in excel (data.xlsx), and a short instructional document about running the model (Read me.pdf).
(ZIP)
