# Supplementary material for: Late retirement, early careers, and the aging of U.S. science and engineering professors
Source: PLoS One. 2018 Dec 26;13(12):e0208411. doi: 10.1371/journal.pone.0208411 (PMC6306255; doi:10.1371/journal.pone.0208411)
Supplement: S1 File — The file documents sensitivity analysis of the simulation model. (DOCX) [file pone.0208411.s001.docx]

**S1 File.** Sensitivity analysis.

Like any model, ours has a few assumptions, and therefore all results should be interpreted with caution. The focus of the model is mainly on the physics of the population flow as they age, thus behavioral assumptions are minimal in our model, which makes it relatively robust.

The model mainly follows the model structure in Blau and Weinberg (2017) with the main difference being hiring. Specifically, our assumption is that any one exit (retirement or early quitting) will result in one opening. Some may argue that the number might be around one, a much smaller or much bigger number, or a non-linear relation. The Table below lists four potential assumptions. We would like to specifically clarify that even if one does not model the effect of the exit rate on hiring (as per Blau and Weinberg), the practice is equivalent to assuming that, for every one exit, there are zero new hires. In a sense, all models are assuming that for every one exit there is *n* new hires, but they may differ in the value they pick for *n*.

**Table A. Possible Assumptions of the Effect of the Exit Rate on Hiring**

| Potential hiring assumptions | Use | Comment |
| --- | --- | --- |
| 1. Hiring is mainly a function of supply.  For every one exit, there is zero hiring. | Blau and Weinberg (2017) | **Potential justification:** It would be imprecise to model the effects of the exit rate on hiring.  **Criticism:** By not modeling the link, we are not solving the imprecision issue. However, we are assuming that the effect is zero, which might be even more imprecise than assuming any other positive number around 1. |
| 2. Hiring is mainly a function of demand (constraint being enough supply).  For every one exit, there is one hiring. | This paper | **Potential justification:** Universities react to losses in their workforce because they must meet a level of teaching, advising, and research workload. Furthermore, universities react to their expertise gaps. For example, a public policy department that loses its economics professor tries to replace him/her with a new economics professor.  **Criticism:** This implies that no sensitivity exists to a salary difference and experience difference between a retiree and a new employee. |
| 3. Hiring is a function of resources.  For every one exit, there is more than one hiring. | Not yet | **Potential justification:** Universities free more of their budget when one highly established person retires, and more than one junior faculty could be potentially hired as replacement.  **Criticism:** The difference between salaries is not much when one thinks about an aggregate-level university budget; The university may use the remaining resource for other purposes. |
| 4. Hiring is a function of desired output (e.g., total teaching hours per year).  For every one exit, there is less than one hiring. | Not yet | **Potential justification:** Universities can expect more productivity from new and young faculty (e.g., papers/years), thus with a less-productive person leaving, they can perform the expected job with less than one person.  **Criticism:** Universities do not necessarily have a target for research and teaching, and with more faculty members they can simply decrease course loads. |

Comparing the four assumptions, we still believe that the second assumption, which is implemented in this paper, is simple and reasonable (Schillebeeckx, Maricque, & Lewis, 2013). However, we conduct a sensitivity analysis and simulate the model for a wide range of scenarios with two extremes of a full exogenous hiring (similar to Blau and Weinberg’s model) and a full endogenous model (1 hire for every 1 exit), as well as in-between scenarios (the weighted average of two hiring models; w=0 full exogenous hiring, w=1 full endogenous hiring). Figure below presents the results. The exogenous hiring model explains 44% of the change in average age in data from 1995 to 2010, and the full endogenous model explains 65% of the change. Please note that these results are not easily comparable with the 8% estimation of Blau and Weinberg, as they analyzed the entire science and engineering workforce with no capacity limit, and their estimation is for the 1993–2008 period.

**Fig A.** The sensitivity analysis. In the full exogenous hiring formulation, 44% of the change in average age between 1995 and 2010 is explained by late retirement. In the endogenous model with one hire for every one exit, the model explains 65% of the change in average age.

References

Ashenfelter, O., & Card, D. (2002). Did the elimination of mandatory retirement affect faculty retirement?. American Economic Review, 92(4), 957-980.

Blau, D. M., & Weinberg, B. A. (2017). Why the US science and engineering workforce is aging rapidly. Proceedings of the National Academy of Sciences, 114, 3879-3884.
