# Supplementary material for: Late retirement, early careers, and the aging of U.S. science and engineering professors
Source: PLoS One. 2018 Dec 26;13(12):e0208411. doi: 10.1371/journal.pone.0208411 (PMC6306255; doi:10.1371/journal.pone.0208411)
Supplement: S2 File — This file includes an additional simulation analysis depicting the future aging trends. (DOCX) [file pone.0208411.s002.docx]

**S2 File.** Future trends analysis.

We further analyze the model by projecting future age trends. We run the simulation model with the given parameters until 2025. On the demand side, we need to make an assumption about university capacities. The current growth trend in faculty positions is on the order of 1.3% annual growth. We run our model under four different scenarios in regards to university capacities: constant faculty slots (0% growth rate), and 1%, 2%, and 3% annual growth rates. In the scenario of constant faculty slots, hiring only occurs to replace the exit rate. In other scenarios, in addition to replacement, universities are increasing their tenure-track positions and thus hiring more new faculty members.

Figure below shows such results for all four scenarios. In the most reasonable scenarios of 1%–2% annual growth, the average age of faculty members is predicted to reach a steady state of 50.9–51.7 years in 2025.

**Fig A.** The average age trend until 2025 under four different scenarios in regards to university capacity (constant faculty slots and 1%, 2%, and 3% annual growth).
