# Supplementary material for: Late retirement, early careers, and the aging of U.S. science and engineering professors
Source: PLoS One. 2018 Dec 26;13(12):e0208411. doi: 10.1371/journal.pone.0208411 (PMC6306255; doi:10.1371/journal.pone.0208411)
Supplement: S3 File — The file includes different conceptual models of aging of university professors with varying complexity. (DOCX) [file pone.0208411.s003.docx]

**S3 File.** Conceptual models of aging of university professors.

Conceptual maps of aging faculty members with different levels of details are presented in this Appendix. Fig A shows the common linear approach in previous studies. Fig B is a simple conceptual representation of our model. Fig C shows potential ways to extend our model in future studies.

**Fig A.** A simple linear representation of *tenure-track or tenured professors* as affected by inflow and outflows.

Note: This is the structure represented in more details in previous studies.

**Fig B.** An extension of Fig A; a representation of the stock of *tenure-track or tenured professors* as affected by inflows and outflows and influences them.

Note: This is a simple representation of the structure of the model presented in this paper.

- Loops B1, B2, and B3: As professors leave (due to various reasons of tenure denial, attrition and move to industry, or retirement) – everything else kept constant – we have fewer professors, and fewer exit rate.
- Loops B4 and R1: hiring rate depends on exit rate and capacity gap. With more capacity gap we hire more which closes the gap; with more exit rate, we hire more to fill the available positions.

**Fig C**. Potential additional dynamic hypotheses for future studies.

Note: Loops:

- R2: Professors train PhD students a portion of whom become future professors.
- R3: Postdocs and other scholars in non-tenure positions apply for tenure-track jobs. More applicants make the market more competitive leading to more people not landing tenure-track positions who end up taking postdoc positions.
- R4: Postdocs help professors which leads to training more PhD students.
- R5: More competition leads to higher quality hires in academia. Higher quality hires have higher likelihood of getting tenure leading to less exit rate, fewer openings, and even more competition.
- B5: Higher market competitiveness and higher bargaining power of universities may lead to increasing tenure standards which increases exit rate.
- R6 and R7: Higher quality new professors and established professors lead to higher average productivity in academia and less exit rate and openings.
- R8: Growing institutional incentive for late retirement due to competitive advantage of experienced professors in getting funding.
- R9: With higher average retirement age, the fraction of faculty who are in tenure-track declines, which leads to overall less openings due to tenure-denial, and less openings.
- B7: Less productivity leads to retirement.
- B8: Natural limits and personal decisions regarding working in old ages.
- B9: Institutional incentives for decreasing retirement age (such as offering retirement packages).
