# Supplementary material for: Late retirement, early careers, and the aging of U.S. science and engineering professors
Source: PLoS One. 2018 Dec 26;13(12):e0208411. doi: 10.1371/journal.pone.0208411 (PMC6306255; doi:10.1371/journal.pone.0208411)

Read me – Instruction for working with the simulation model.

Steps to run the model

1- Unzip the folder. If you don't unzip the folder you may later receive an error in simulation.
2- Make sure the data file (excel) is in the same folder as the simulation file (vensim).
3- Open the Vensim file with Vensim DSS. In order to Run the model, you need Vensim DSS (not Vensim PLE).

Your model dashboard will look like this, after running the model (preferably run by clicking on the Synthesim button on the top of your screen):

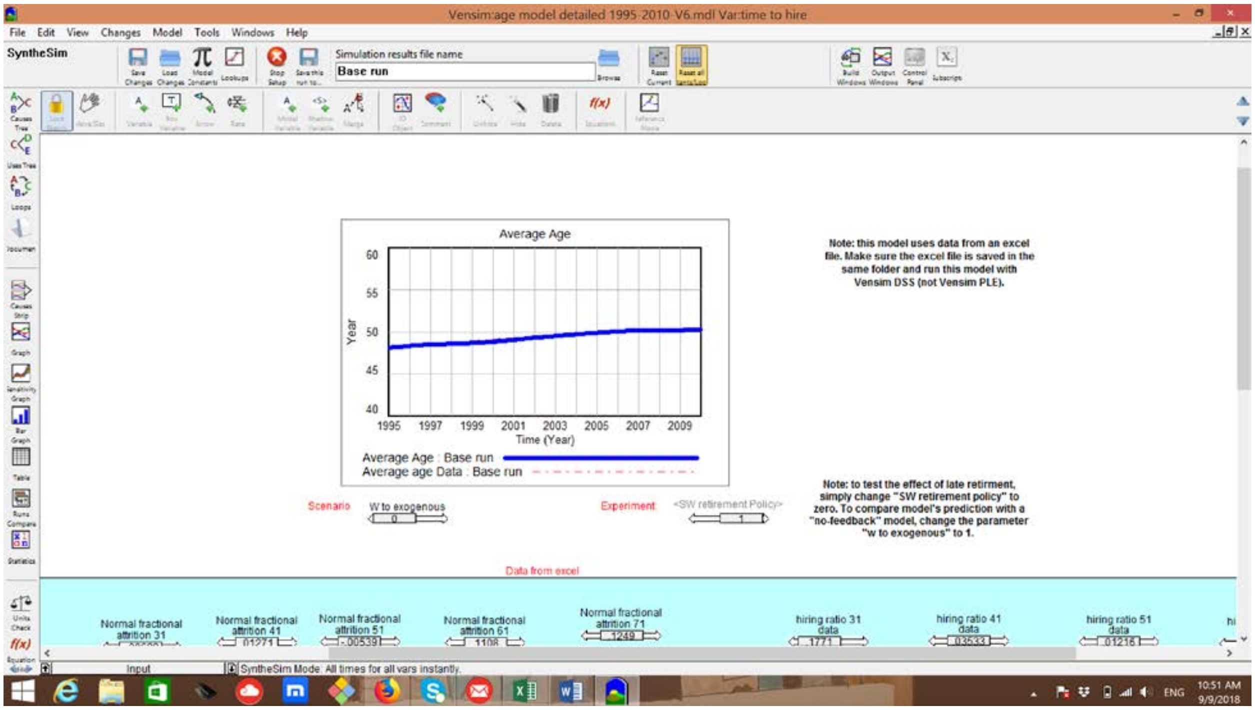

Supplement: S4 File — The zipped folder includes the simulation model in Vensim DSS (AgeModel-V6.mdl), the data file for the simulation model in excel (data.xlsx), and a short instructional document about running the model (Read me.pdf). (ZIP) [file pone.0208411.s004.zip › Model and data/Read me.pdf]
